# Outcomes of patients with initially unresectable pancreatic cancer who underwent conversion surgery after FOLFIRINOX or gemcitabine plus nab‐paclitaxel chemotherapy: A multicenter retrospective cohort study (PC‐CURE‐1)

**DOI:** 10.1002/jhbp.12066

**Published:** 2024-08-16

**Authors:** Naohiro Okano, Manabu Kawai, Makoto Ueno, Xianjun Yu, Yosuke Inoue, Shinichiro Takahashi, Wenquan Wang, Hidenori Takahashi, Yukiyasu Okamura, Soichiro Morinaga, Ippei Matsumoto, Yasuhiro Shimizu, Kazuhiro Yoshida, Tomohisa Yamamoto, Masayuki Ohtsuka, Yoshikuni Inokawa, Satoshi Nara, Jun Tamura, Satoru Shinoda, Kouji Yamamoto, Hiroki Yamaue, Junji Furuse, PC‐CURE‐1 study group

**Affiliations:** ^1^ Department of Medical Oncology Kyorin University Faculty of Medicine Tokyo Japan; ^2^ Second Department of Surgery Wakayama Medical University School of Medicine Wakayama Japan; ^3^ Department of Gastroenterology Kanagawa Cancer Center Yokohama Japan; ^4^ Department of Pancreatic Surgery Fudan University Shanghai Cancer Center Shanghai China; ^5^ Division of Hepatobiliary and Pancreatic Surgery Cancer Institute Hospital, Japanese Foundation for Cancer Research Tokyo Japan; ^6^ Department of Hepatobiliary and Pancreatic Surgery National Cancer Center Hospital East Kashiwa Japan; ^7^ Department of Gastroenterological Surgery Osaka International Cancer Institute Osaka Japan; ^8^ Division of Hepato‐Biliary‐Pancreatic Surgery Shizuoka Cancer Center Hospital Shizuoka Japan; ^9^ Department of Hepato‐Biliary and Pancreatic Surgery Kanagawa Cancer Center Yokohama Japan; ^10^ Department of Surgery Kindai University Faculty of Medicine Osakasayama Japan; ^11^ Department of Gastroenterological Surgery Aichi Cancer Center Hospital Nagoya Japan; ^12^ Department of Surgical Oncology Gifu University Graduate School of Medicine Gifu Japan; ^13^ Department of Surgery Kansai Medical University Hirakata Japan; ^14^ Department of General Surgery, Graduate School of Medicine Chiba University Chiba Japan; ^15^ Department of Gastroenterological Surgery Nagoya University Graduate School of Medicine Nagoya Japan; ^16^ Department of Hepatobiliary and Pancreatic Surgery National Cancer Center Hospital Tokyo Japan; ^17^ Department of Biostatistics Yokohama City University School of Medicine Yokohama Japan

**Keywords:** albumin‐bound paclitaxel, FOLFIRINOX, gemcitabine, pancreatic cancer, surgery

## Abstract

**Background:**

The efficacy and safety of conversion surgery (CS) after FOLFIRINOX or gemcitabine plus nab‐paclitaxel (GnP) chemotherapy in patients with initially unresectable pancreatic cancer (PC) remains unclear.

**Methods:**

This multicenter retrospective cohort study enrolled patients, between 2014 and 2018, with initially locally advanced or metastatic PC who were considered candidates for CS following FOLFIRINOX or GnP chemotherapy. They were classified into surgery (207 patients [194 resection and 13 exploratory laparotomy only]) and continued chemotherapy (10 patients, control) groups. The primary endpoint was overall survival (OS) from the day of diagnosis of potentially curative resection on imaging studies, with an expected hazard ratio (HR) of 0.7.

**Results:**

OS in the surgery group was longer than that in the control group (HR, 0.47; 95% confidence interval [CI]: 0.24–0.93). The median OS was 34.4 (95% CI: 27.9–43.4) and 19.8 (95% CI: 14.9–31.1) months in the surgery and control groups, respectively. The Clavien‐Dindo grade ≥ IIIa postoperative complication and in‐hospital mortality rates were 19.6% and 0.5%, respectively. Multivariate analysis revealed that preoperative chemotherapy duration was not associated with OS.

**Conclusions:**

CS, following a favorable response to FOLFIRINOX or GnP chemotherapy, improved initially unresectable PC prognosis (specifically, OS), regardless of the chemotherapy duration.

## Graphical abstract

Okano and colleagues aimed to clarify the outcomes, safety, and prognostic factors of patients with initially unresectable pancreatic cancer who underwent conversion surgery after FOLFIRINOX or gemcitabine plus nab‐paclitaxel chemotherapy. Conversion surgery, following a favorable response to chemotherapy, improved initially unresectable pancreatic cancer outcomes, regardless of the duration of chemotherapy.

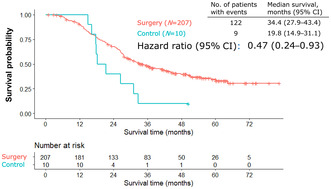

## 1 INTRODUCTION

Despite rapid advances in cancer therapy, the prognosis of patients with pancreatic cancer (PC) remains extremely poor, with a 5‐year survival rate of approximately 10%.^1^ PC is classified into resectable, borderline resectable, locally advanced, and distant metastasis stages. Currently, only surgical resection followed by adjuvant chemotherapy leads to cure or long‐term survival; however, only 15%–20% of patients with PC have resectable lesions at the time of diagnosis.^1^, ^2^ Borderline resectable, unresectable locally advanced, and metastatic PCs are initially treated with chemotherapy or chemoradiotherapy.

In 2010s, two phase III trials demonstrated the superiority of 5‐fluorouracil, leucovorin, irinotecan, oxaliplatin (FOLFIRINOX), and gemcitabine plus nab‐paclitaxel (GnP) over gemcitabine monotherapy in patients with metastatic PC. FOLFIRINOX and GnP have marked antitumor effects, with response rates of 31.6% and 23%, respectively.^3^, ^4^ Additionally, both regimens are widely used in patients with unresectable locally advanced PC because of their high response rates and survival benefits compared with regimens such as gemcitabine monotherapy or chemoradiotherapy. The number of unresectable PCs that become resectable via local control or downstaging is increasing with advancements in these chemotherapies.^5^

According to current consensus, conversion surgery (CS) is defined as radical resection following chemotherapy and/or chemoradiotherapy in patients with initially unresectable locally advanced or metastatic PC.^6^ Its effectiveness against locally advanced PC has been examined.^5^, ^7^ Moreover, even patients with distant metastases may be cured or have an increased survival rate post‐primary lesion excision if the distant metastases “disappeared.”^8^ Previous studies on long‐term survival outcomes of CS after FOLFIRINOX or GnP chemotherapy have compared patients who underwent CS with patients who were not candidates for CS.^9^, ^10^, ^11^, ^12^ Thus, the efficacy of CS remains unclear for patients with a good response to chemotherapy in whom unresectable PC becomes potentially resectable after FOLFIRINOX or GnP chemotherapy. Moreover, the current knowledge on the morbidity and mortality rates of CS after FOLFIRINOX or GnP chemotherapy is limited. Therefore, this study aimed to clarify the prognosis, safety, and prognostic factors of patients with initially unresectable PC who underwent CS after FOLFIRINOX or GnP chemotherapy.

## 2 METHODS

### 2.1 Study design, setting, and participants

This multicenter, retrospective cohort study, conducted by the participating institutions of the Federation of Asian Clinical Oncology in China, South Korea, and Japan, enrolled patients with potentially resectable, initially locally advanced or metastatic PC who became candidates for CS based on radiological findings after a sufficient duration of FOLFIRINOX or GnP chemotherapy. The participants were classified into two groups: those who underwent laparotomy (surgery group) and those who continued chemotherapy (control group). The surgery group included patients who underwent CS or exploratory laparotomy only. The control group included patients who were deemed to have potentially resectable PC during chemotherapy but did not undergo laparotomy owing to physician discretion or patient's refusal and those for whom CS could have been recommended on retrospective scrutiny. Patients with initially unresectable PC deemed to have converted to potentially resectable PC after FOLFIRINOX or GnP chemotherapy between January 1, 2014, and December 31, 2018, were registered in a data center between February 1, 2019, and January 31, 2021.

### 2.2 Data collection

First, the following patients' background information was collected before chemotherapy induction: age, sex, Eastern Cooperative Oncology Group performance status, extent of disease, tumor location, primary tumor size, presence or absence of biliary drainage, reasons why resection was impossible, tumor, node, metastasis (TNM) staging according to the Union for International Cancer Control (UICC) version 7, and tumor markers including carcinoembryonic antigen (CEA) and carbohydrate antigen 19‐9 (CA 19‐9). Second, the following preoperative information was collected: chemotherapy duration, prior or no radiotherapy, duration to surgery from last chemotherapy administration, response to chemotherapy according to Response Evaluation Criteria in Solid Tumors (RECIST) version 1.1.,^13^ presence or absence of biliary drainage, TNM staging according to the UICC version 7, presence or absence of arterial and portal vein invasion, and CEA and CA 19‐9. Third, the following operative findings were collected: operation type, combined resection of structures/organs, R status, TNM staging according to the UICC version 7, response to chemo(radio)therapy according to the Evans grading system,^14^ and postoperative mortality or in‐hospital death and morbidity according to Clavien–Dindo grade ≥ IIIa.^15^ Fourth, data on the postoperative adjuvant therapy regimens were collected.

### 2.3 Inclusion and exclusion criteria

The inclusion criteria were as follows:(1) histologically initially confirmed or cytologically proven pancreatic adenocarcinoma or adenosquamous carcinoma and subsequently via diagnostic imaging; (2) unresectable locally advanced or metastatic PC before chemotherapy induction; (3) initial treatment with FOLFIRINOX (including the modified regimen) or GnP; and (4) diagnosed with potentially resectable tumor post‐chemotherapy via imaging, and subsequently, underwent laparotomy or continued chemotherapy.

In this study, CS was performed when patients were comprehensively diagnosed with potentially resectable tumor post‐chemotherapy via imaging, tumor makers, and other criteria: (1) radiological examination indicated a complete response, partial response, or stable disease according to the RECIST version 1.1; (2) metastatic lesions disappeared on radiological examination after chemotherapy in patients with initially distant metastatic disease; (3) tumor markers such as serum CA 19‐9 or CEA decreased considerably; (4) no new metastatic sites appeared; (5) Eastern Cooperative Oncology Group performance status was maintained at 0–1; and (6) curative resection was technically possible for the primary pancreatic tumor.

Conversely, for unresectable tumors, curative resection was considered infeasible in the following cases:^16^, ^17^, ^18^ (1) need for superior mesenteric artery resection due to superior mesenteric artery involvement, (2) solid tumor with encasement (>180° contact) of the celiac artery, (3) reconstruction was infeasible despite need for concomitant resection of the common hepatic artery and/or proper hepatic artery due to artery infiltration, (4) constructible superior mesenteric vein/portal vein was infeasible despite need for portal vein concomitant resection, or (5) tumor marker response was poor after chemotherapy.

The exclusion criteria were as follows: (1) borderline resectable PC according to the National Comprehensive Cancer Network (NCCN) guidelines version 2.2018, (2) solid tumor contact with the celiac artery >180°, (3) recurrent disease, (4) progressive disease (response to chemotherapy according to RECIST version 1.1), (5) treatment with prelaparotomy heavy ion or proton beam radiotherapy, (6) diagnosis of unresectable tumor on laparoscopic examination when CS was intended, (7) resection of metastatic lesions during CS (registration was not allowed unless patients were pathologically confirmed to have cancer after resection [no. 16 lymph node] or hepatectomy), and (8) positive peritoneal lavage cytology at CS.

### 2.4 Ethical considerations

This study was conducted in accordance with the Declaration of Helsinki guidelines and was approved by the Ethics Committee of the Kyorin University Faculty of Medicine (approval no. 743) and each participating institution. Informed consent was not obtained from the patients because of the retrospective study design. Nevertheless, the opt‐out option was offered to patients who wished to refuse to participate. This study was registered with the UMIN Clinical Trials Registry (UMIN000035668). A follow‐up survey on prognosis was conducted 6 months after registration completion.

### 2.5 Chemotherapy or chemoradiotherapy

The selection of FOLFIRINOX or GnP and duration of chemotherapy depended on the physician's discretion. Initial dose modification of FOLFIRINOX and GnP chemotherapy was permitted. Dose modification after chemotherapy induction was performed at the physician's discretion. Combined conventional radiotherapy (concurrent or sequential) was permitted.

### 2.6 Surgical procedure and adjuvant therapy

No particular surgical procedure or adjuvant therapy was prescribed, because this was a retrospective observational study. The regimen and duration of adjuvant therapy were based on physician discretion. The pathological findings for response to chemotherapy or chemoradiotherapy were evaluated according to the Evans grading system. The evaluation of the Evans grading system was not collected from China because they were not evaluated in clinical practice, and this information was mentioned in the study protocol.

### 2.7 Endpoints

The primary endpoint was overall survival (OS) from the day when the initially unresectable PC was diagnosed as potentially curative resection by imaging (date of imaging). The secondary endpoints were relapse‐free survival (RFS), post‐resection survival (PRS), mode of relapse, presence or absence of in‐hospital death, and surgery‐related complications.

### 2.8 Date of imaging deemed as potentially resectable PC

The interval of computed tomography or magnetic resonance imaging was based on each institute's policy. The following criteria were applied to the imaging data to be deemed as potentially resectable PC: (1) for the surgery group, the date of diagnostic imaging immediately before scheduled laparotomy was regarded as the date when resection was deemed possible; and (2) for the control group, the date of diagnostic imaging by which CS was regarded as possible (for patients who abandoned CS because of physician discretion or refusal), or the date of diagnostic imaging on which resection could have been recommended based on imaging findings (for patients included based on retrospective scrutiny) was regarded as the date when resection could have been performed.

### 2.9 Statistical analysis

The OS was defined as the time from the date of imaging to the date of death from any cause. The RFS and PRS were measured as the time from surgery to the date of disease progression or death from any cause, whichever occurred earlier. Survival curves were estimated using the Kaplan–Meier method. Additionally, the prognostic factors involved in OS were examined via multivariate analyses using the Cox proportional hazards regression model.

In a previous retrospective study, the mean OS after chemotherapy induction (FOLFIRINOX) in patients with locally advanced PC who underwent CS was approximately 30 months.^7^ The ratio of patients with locally advanced PC to those with metastatic PC was assumed to be 9:1. Therefore, the median OS of patients who underwent CS in the present study was estimated to be approximately 30 months. Even if more metastatic PC cases were registered, as in a previous study where the median OS calculated from the date of diagnosis in patients who underwent resection of the primary lesion after the “disappearance” of distant metastasis by chemotherapy was 56 months,^8^ the difference was considered insignificant.

Assuming a median interval of 6 months from chemotherapy induction to CS, the median OS was estimated to be approximately 24 (i.e., 30–6) months, considering the day when CS was deemed possible as the starting point. If the hazard ratio (HR) were 0.7 (24.0 months in surgery group vs. 16.8 months in control group), the median OS would have been prolonged by 7.2 months. Therefore, for a minimum clinically important improvement corresponding to the invasiveness of the CS, an HR of 0.7 was targeted.

Regarding the surgery group, based on the results of a questionnaire survey, approximately 150–200 patients treated with resection/conversion surgery were speculated to register in Japan, in contrast to only few such patients in China. Therefore, the number of patients undergoing surgery was estimated to be ≥200. Regarding the control group, 20–30 Japanese patients (patients who refused surgery despite its possibility) were speculated to register. A pre‐study investigation revealed that conversion surgery was not performed for such patients in clinical practice in Korea (5 institutions); therefore, approximately 100 patients were speculated to register. Thus, the number of controls was estimated to be ≥100.

The number of events required to find a hazard ratio of 0.7 under a paired significance level (paired α) of 5% and detection power of 80% was 278 deaths (events) at a 2:1 (surgery: control groups) allocation. Since the study aimed to observe approximately 300 events at this ratio, the target numbers of patients in the surgery and control groups were established as 240 and 120, respectively. All statistical tests were two‐sided, and *p*‐values < .05 were considered statistically significant. Statistical analyses were performed using the R software version 4.0.2 (R Foundation, Boston, MA, USA).

## 3 RESULTS

### 3.1 Patients

**FIGURE 1:**
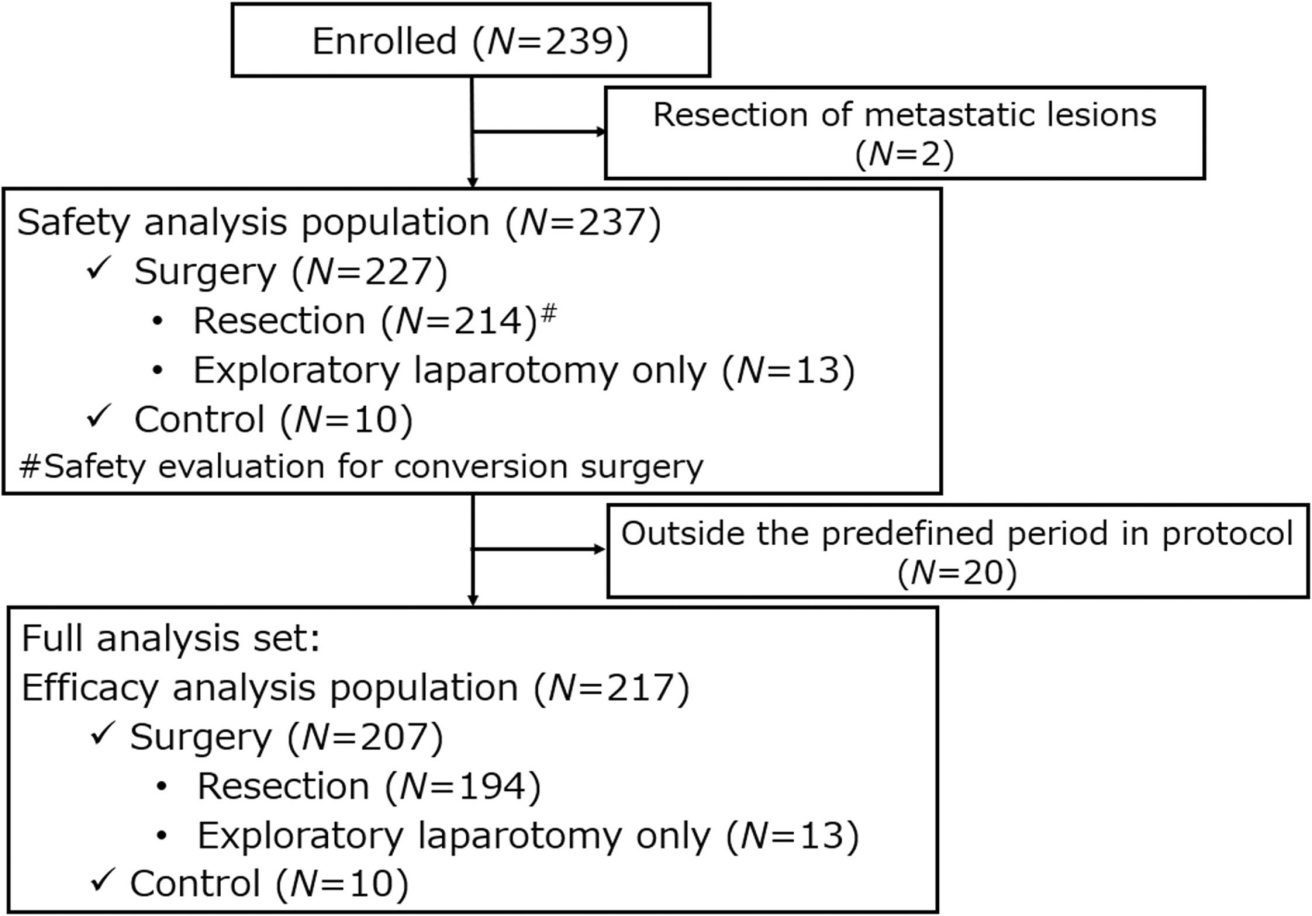
Patient flow chart.

A patient flow chart is shown in Figure 1. Among the 239 patients enrolled from 35 institutions, 207 patients (194 resections and 13 exploratory laparotomies) and 10 patients in the surgery and control groups were included, respectively.

The prechemotherapy and preoperative patient characteristics are listed in Tables 1 and 2, respectively. Duration from last chemotherapy cycle to surgery differed between the resection and exploratory laparotomy only groups. Specifically, the proportion of patients with ≥4 weeks between the decision of potentially resectable PC and laparotomy was 56.2% in the resection group and 100% in the exploratory laparotomy only group. Operative information of the 194 patients who underwent resection is listed in Table 3. A summary of 13 exploratory laparotomies is shown in Table S1.

**TABLE 1:** Prechemotherapy patient characteristics and information on chemotherapy duration and radiotherapy.

Characteristics	Surgery (*N* = 207)	Control (*N* = 10)
Sex		
Male/female, *N* (%)	111/96 (53.6/46.3)	5/5 (50.0/50.0)
Age, years		
Median (IQR)	65.0 (60.0–69.5)	68.5 (64.5–73.0)
<65 years/≥65 years, *N* (%)	90/117 (43.5/56.5)	3/7 (30.0/70.0)
ECOG PS		
0/1/2, *N* (%)	179/26/2 (86.5/12.6/1.0)	9/1/0 (90.0/10.0/0)
Country		
Japan/China, *N* (%)	202/5 (97.6/2.4)	10/0 (100/0)
Extent of disease		
Locally advanced/metastatic, *N* (%)	136/71 (65.7/34.3)	8/2 (80.0/20.0)
Tumor location		
Head/body or tail, *N* (%)	118/89 (57.0/43.0)	7/3 (70.0/30.0)
Tumor diameter, mm		
Median (IQR)	30.0 (24.0–38.0)	32.0 (30.3–35.8)
Regional lymph node metastasis		
Yes/no, *N* (%)	60/147 (29.0/71.0)	5/5 (50.0/50.0)
Biliary drainage		
Yes/no, *N* (%)	71/136 (34.3/65.7)	2 (20.0/80.0)
Unresectability factors, *N* (%)		
Arterial invasion	147 (71.0)	7 (70.0)
Portal vein invasion	104 (50.2)	8 (80.0)
Liver metastasis	44 (21.3)	2 (20.0)
Lung metastasis	3 (1.4)	0
Lymph node metastasis	16 (7.7)	1 (10.0)
Peritoneal metastasis	14 (6.8)	1 (10.0)
Ascites	1 (0.5)	0
Pleural metastasis	1 (0.5)	0
Others	2 (1.0)	1 (10.0)
TNM by UICC (version 7), *N* (%)		
Stage IIA	14 (6.8)	3 (30.0)
Stage IIB (T3 + N1 + M0)	10 (4.8)	0
Stage III	112 (54.1)	5 (50.0)
Stage IV	71 (34.3)	2 (20.0)
CEA, ng/mL		
Median (IQR)	3.3 (2.0–5.9)	3.25 (1.9–4.7)
Normal (≤5.0 ng/mL), *N* (%)	134 (64.7)	9 (90.0)
Abnormal (>5.0 ng/mL), *N* (%)	61 (29.5)	1 (10.0)
Not available	12 (5.8)	0
CA 19‐9, U/mL		
Median (IQR)	242.0 (49.0–1005.2)	289.7 (59.7–584.0)
Normal (≤37.0 U/mL), *N* (%)	45 (21.7)	2 (20.0)
Abnormal (>37.0 U/mL), *N* (%)	160 (77.3)	8 (80.0)
Not available	2 (1.0)	0
Chemotherapy, *N* (%)		
FOLFIRINOX	53 (25.6)	2 (20.0)
GnP	154 (74.4)	8 (80.0)
Chemotherapy duration, months		
Median (IQR)	6.9 (4.6–9.9)	6.2 (4.4–10.2)
Radiotherapy, *N* (%)		
Performed		
Concurrent	19 (9.2)	1 (10.0)
Sequential	18 (8.7)	1 (10.0)
None	170 (82.1)	8 (80.0)

Abbreviations: CA 19‐9, serum carbohydrate antigen 19‐9; CEA, serum carcinoembryonic antigen; ECOG PS, Eastern Cooperative Oncology Group performance status; FOLFIRINOX, 5‐fluorouracil, leucovorin, irinotecan, and oxaliplatin; GnP, gemcitabine plus nab‐paclitaxel; IQR, interquartile range; TNM, tumor, node, metastasis; UICC, Union for International Cancer Control.

**TABLE 2:** Preoperative patient characteristics.

Characteristics	Resection (*N* = 194)	Exploratory laparotomy only (*N* = 13)
Chemotherapy duration, months		
Median (IQR)	6.9 (4.5–9.8)	7.7 (5.1–13.2)
<6 months/≥6 months, *N* (%)	79/115 (40.7/59.3)	5/8 (38.5/61.5)
<8 months/≥8 months, *N* (%)	117/77 (60.3/39.7)	7/6 (53.8/46.2)
Duration to surgery from last chemotherapy cycle		
<4 weeks/≥4 weeks, *N* (%)	85/109 (43.8/56.2)	0/13 (0/100)
Response to chemotherapy		
CR/PR/SD, *N* (%)	2/146/46 (1.0/75.3/23.7)	0/9/4 (0/69.2/30.8)
Radiotherapy		
None/done, *N* (%)	158/36 (81.8/18.2)	12/1 (92.3/7.7)
Regional lymph node metastasis		
Yes/no, *N* (%)	34/160 (17.5/82.5)	1 (7.7/92.3)
Biliary drainage		
Yes/no, *N* (%)	68/126 (35.1/64.9)	3/10 (23.1/76.9)
TNM by UICC (version 7), *N* (%)		
Stage IA	9 (4.6)	1 (7.7)
Stage IB	1 (0.5)	0 (0.0)
Stage IIA	80 (41.2)	5 (38.5)
Stage IIB (T3 + N1 + M0)	23 (11.9)	1 (7.7)
Stage III	81 (41.8)	6 (46.2)
Arterial invasion		
Yes/no, *N* (%)	90/104 (46.4/53.6)	10/3 (76.9/23.1)
Portal vein invasion		
Yes/no, *N* (%)	77/117 (39.7/60.3)	8/5 (61.5/38.5)
CEA, ng/mL		
Median (IQR)	2.9 (2.1–4.2)	2.5 (2.1–3.2)
Normal (≤5.0 ng/mL), *N* (%)	167 (86.1)	10 (76.9)
Abnormal (>5.0 ng/mL), *N* (%)	27 (13.9)	2 (15.4)
Not available	0	1 (7.7)
CA 19‐9, U/mL		
Median (IQR)	21.0 (8.6–50.0)	36.0 (25.4–68.2)
Normal (≤37.0 U/mL), *N* (%)	137 (70.6)	6 (46.2)
Abnormal (>37.0 U/mL), *N* (%)	57 (29.4)	6 (46.2)
Not available	0	1 (7.7)

Abbreviations: CA 19‐9, serum carbohydrate antigen 19‐9; CEA, serum carcinoembryonic antigen; CR, complete response; IQR, interquartile range; PR, partial response; SD, stable disease; TNM, tumor, node, metastasis; UICC, Union for International Cancer Control.

**TABLE 3:** Operative information (*N* = 194).

Operation type, *N* (%)	
PD/DP/TP/DP‐CAR	113/37/5/39 (58.2/19.1/2.6/20.1)
Combined resections of other structures/organs, *N* (%)	
None	80 (41.2)
Common hepatic artery	13 (6.7)
Celiac artery	32 (16.5)
SMA	0
PV/SMV	77 (39.7)
Liver	0
Colon	6 (3.1)
Adrenal	20 (10.3)
Others	19 (9.8)
Pathological findings	
R status, *N* (%)	
R0/R1/R2	178/14/2 (91.8/7.2/1.0)
TNM by UICC (version 7), *N* (%)	
Stage 0	3 (1.5)
Stage IA	20 (10.3)
Stage IB	5 (2.6)
Stage IIA	79 (40.7)
Stage IIB (T1, T2 + N1 + M0)	10 (5.2)
Stage IIB (T3 + N1 + M0)	55 (28.4)
Stage III	14 (7.2)
No residual cancer	8 (4.1)
Evans grading system, *N* (%)	
I/IIa/IIb/III/IV/NA	32/68/46/31/12/5 (16.5/35.1/23.7/16.0/6.2/2.6)
Postoperative mortality and morbidity, *N* (%)	
In‐hospital mortality, *N* (%)	1 (0.5)
Clavien–Dindo grade ≥ IIIa, *N* (%)	41 (21.1)
Adjuvant therapy, *N* (%)	
S‐1	117 (60.3)
Gemcitabine	7 (3.6)
Gemcitabine plus capecitabine	25 (12.9)
Capecitabine	1 (0.5)
Others	8 (4.1)
None	36 (18.6)

Abbreviations: DP, distal pancreatectomy; DP‐CAR, distal pancreatectomy with celiac axis resection; PD, pancreaticoduodenectomy; PV, portal vein; SMA, superior mesenteric artery; SMV, superior mesenteric vein; TNM, tumor, node, metastasis; TP, total pancreatectomy; UICC, Union for International Cancer Control.

### 3.2 Efficacy and safety of CS

**FIGURE 2:**
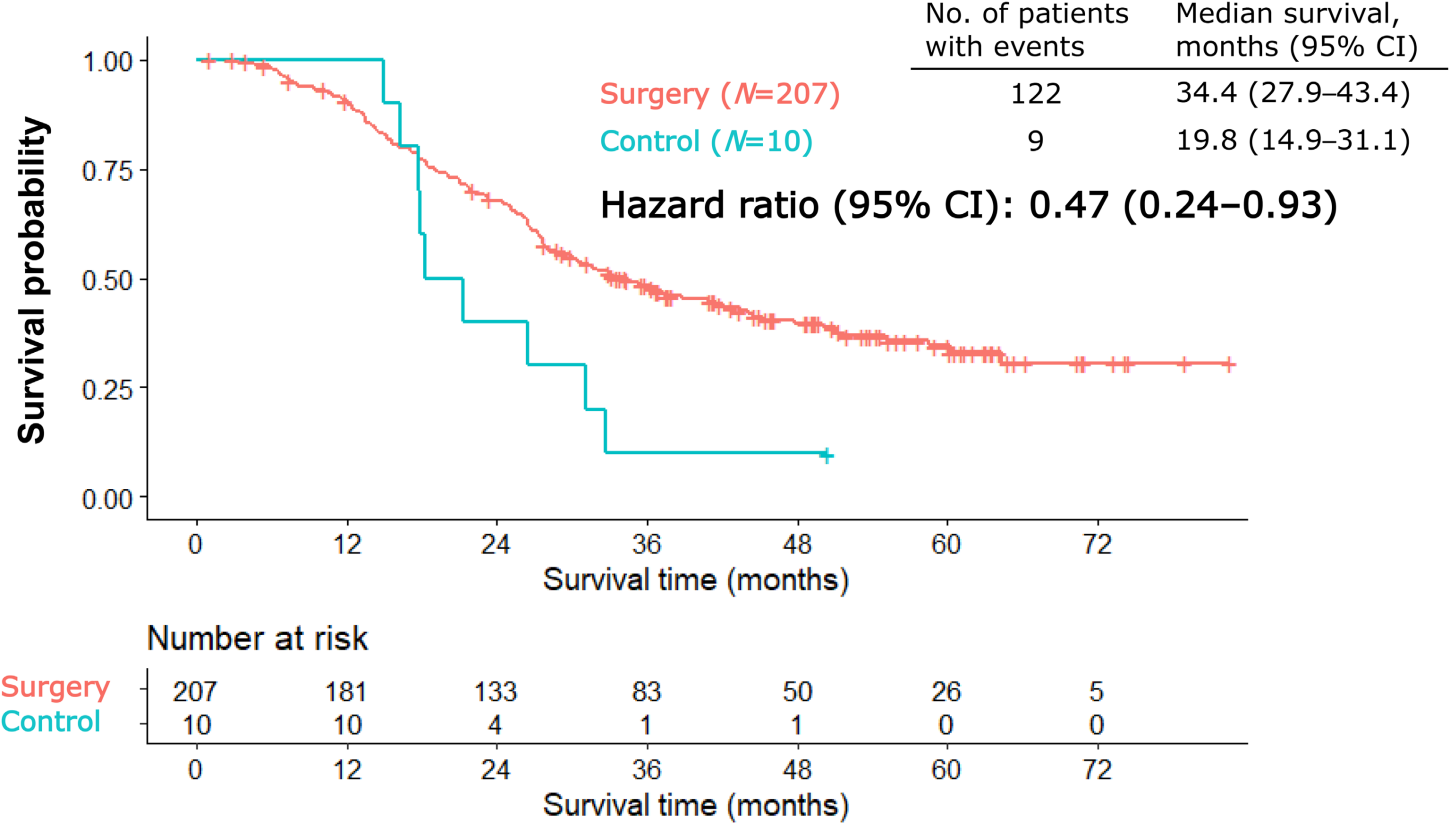
Kaplan–Meier curves of overall survival from the day when initially unresectable pancreatic cancer was determined as potentially curative resection on images. CI, confidence interval.

**FIGURE 3:**
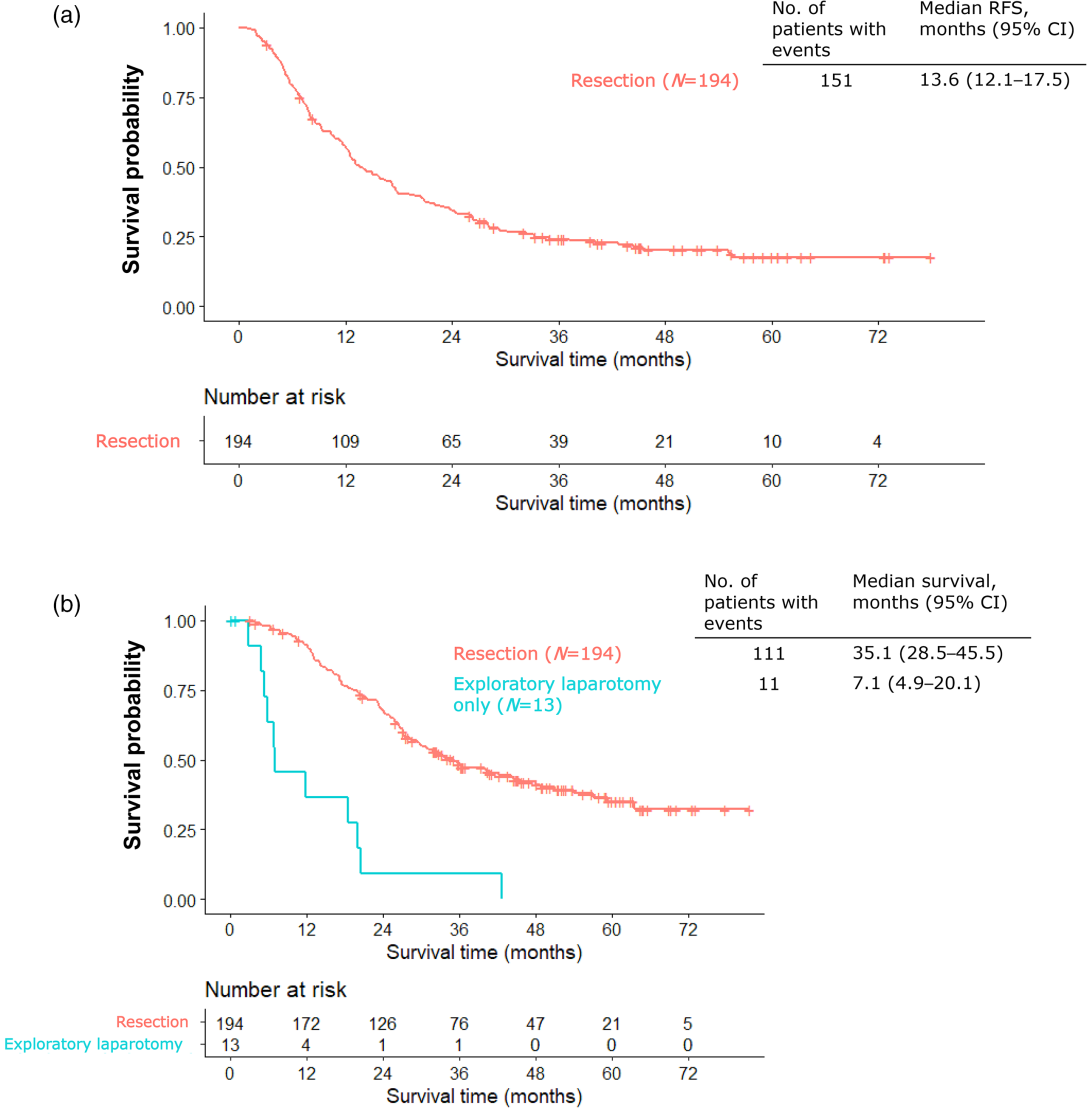
(a) Kaplan–Meier curve for relapse‐free survival. CI, confidence interval; RFS, relapse‐free survival. (b) Kaplan–Meier curves for survival from laparotomy.

Figure 2 illustrates the Kaplan–Meier curves for OS. In the efficacy analysis population, OS was longer in the surgery group than in the control group (HR, 0.47; 95% confidence interval [CI]: 0.24–0.93). The median OS was 34.4 months (95% CI: 27.9–43.4) and 19.8 months (95% CI: 14.9–31.1) in the surgery and control groups, respectively. Figure 3a illustrates the Kaplan–Meier curve for RFS. The median RFS was 13.6 months (95% CI: 12.1–17.5). The Kaplan–Meier curves of time from laparotomy in the resection and exploratory laparotomy only groups are illustrated in Figure 3b. The median PRS was 35.1 months (95% CI: 28.5–45.5). The median survival in the only exploratory laparotomy group was 7.1 months (95% CI: 4.9–20.1). The Kaplan–Meier curves for OS, RFS, and time from laparotomy in the resection and exploratory laparotomy only groups in locally advanced or metastatic PC are illustrated in Figures S1–S6.

Clavien‐Dindo ≥IIIa postoperative complications were observed in 42 (19.6%) of 214 patients who underwent resection in the safety analysis population. Complications included pancreatic fistula (12.6%), intra‐abdominal abscess or infection (8.9%), hemorrhage (2.3%), and bile leakage (0.5%). Four patients (1.9%) required a reoperation. There was one (0.5%) in‐hospital death.

### 3.3 Adjuvant therapy and mode of relapse

Among 194 patients who underwent resection, 158 (81.4%) received adjuvant therapy. The adjuvant therapies used were S‐1 in 117 (60.3%) patients and gemcitabine plus capecitabine in 25 (12.9%) patients. Other therapies included gemcitabine in seven patients (3.6%) and capecitabine in one (0.5%). Among these patients, 51 (26.3%) experienced relapse. The most common sites were the liver (18.0%), local (17.5%), lungs (17.0%), and peritoneum (11.3%).

### 3.4 Multivariate analysis for OS in the surgery group

Multivariate analysis of OS from the date of imaging indicated that preoperative chemotherapy duration (≥6 months vs. <6 months) was not associated with OS (Table 4). As previously stated, this study presupposed a preoperative chemotherapy duration of 6 months. The median preoperative chemotherapy duration in the surgery group was 6.9 months. Therefore, in this multivariate analysis, the preoperative chemotherapy duration was divided using a cutoff of 6 months. Preoperative chemotherapy duration was not associated with OS, even when divided into 4 and 8 months (Tables S2 and S3). Moreover, this finding was the same in analyses stratified by locally advanced or metastatic PC (Tables S4–S9). Furthermore, female sex, FOLFIRINOX, and complete response or partial response according to RECIST version 1.1 were identified as good prognostic factors.

**TABLE 4:** Multivariate analysis of the overall survival of the surgery group divided into 6‐month periods of chemotherapy.

	Hazard ratio (95% CI)	*p*‐value
Sex		
Male	1.58 (1.08, 2.33)	.020
Female	1	
Age		
<65 years	1.30 (0.87, 1.95)	.202
≥65 years	1	
ECOG PS		
0	1.05 (0.61, 1.82)	.855
1 or 2	1	
Extent of disease		
Locally advanced	0.82 (0.45, 1.51)	.523
Metastatic	1	
Tumor location		
Head	0.85 (0.57, 1.26)	.425
Body or tail	1	
Tumor diameter prior to chemotherapy	1.00 (0.98, 1.01)	.964
Regional lymph node metastasis prior to chemotherapy		
Yes	0.90 (0.58, 1.40)	.649
No	1	
Arterial invasion prior to chemotherapy		
Yes	1.05 (0.58, 1.91)	.865
No	1	
Portal vein invasion prior to chemotherapy		
Yes	1.21 (0.79, 1.86)	.373
No	1	
Chemotherapy		
FOLFIRINOX	0.38 (0.22, 0.65)	<.001
GnP	1	
RECIST version 1.1		
CR/PR	0.56 (0.36, 0.88)	.013
SD	1	
Chemotherapy duration		
<6 months	0.75 (0.31, 1.79)	.518
≥6 months	1	
Radiotherapy		
None	1.25 (0.73, 2.17)	.417
Performed	1	
CEA prior to chemotherapy		
Normal	1.12 (0.73, 1.72)	.604
Abnormal	1	
CA 19‐9 prior to chemotherapy		
Normal	0.89 (0.48, 1.64)	.701
Abnormal	1	

Abbreviations: CA 19‐9, serum carbohydrate antigen 19‐9; CEA, serum carcinoembryonic antigen; CI, confidence interval; CR, complete response; ECOG PS, Eastern Cooperative Oncology Group performance status; FOLFIRINOX, 5‐fluorouracil, leucovorin, irinotecan, and oxaliplatin; GnP, gemcitabine plus nab‐paclitaxel; PR, partial response; RECIST, Response Evaluation Criteria in Solid Tumors; SD, stable disease.

### 3.5 Impact of FOLFIRINOX and GnP therapies on CS

The prechemotherapy induction characteristics are listed in Table S10. The median OS was not reached (95% CI: 37.3–not reached) in the FOLFIRINOX group and was 27.9 months (95% CI: 26.1–34.4) in the GnP group (Figure S7). Resection was performed in 51 of 53 (96.2%) patients in the FOLFIRINOX group and 143 of 154 (92.9%) patients in the GnP group. The preoperative characteristics of patients who underwent resection are listed in Table S11. Among these patients, 52.9% and 76.9% in the FOLFIRINOX and GnP groups, respectively, showed arterial invasion. The median RFS and PRS were 28.0 months (95% CI: 19.8–not reached) and not reached (95% CI: 40.1–not reached) in the FOLFIRINOX group, respectively, and 11.6 (95% CI: 8.6–13.6) and 28.3 months (95% CI: 25.3–35.8) in the GnP group, respectively (Figures S8 and S9). Potential differences in the operative information are presented in Table S12. The pathological responses according to Evans grade III or IV were 31.4% and 18.9% in the FOLFIRINOX and GnP groups, respectively.

## 4 DISCUSSION

This was the first study to compare outcomes between surgery and continued chemotherapy in patients with initially unresectable PC deemed as potentially resectable after FOLFIRINOX or GnP chemotherapy. Moreover, this was the largest retrospective cohort study to collect data from patients with initially unresectable locally advanced or metastatic PC who underwent CS after FOLFIRINOX or GnP chemotherapy. The surgery group, including patients who had undergone exploratory laparotomy only, showed improved OS compared to those on continued chemotherapy. Furthermore, the morbidity and mortality rates of CS after FOLFIRINOX or GnP chemotherapy were within the acceptable range. Notably, chemotherapy duration was not associated with OS in the multivariate analysis.

This study demonstrated that the OS and RFS of the surgery group were 34.4 and 13.6 months, respectively. Additionally, the OS of the surgery group was longer than that of the control group (continued chemotherapy) with an HR of 0.47. In this study, with an expected HR of 0.7, CS demonstrated considerable improvement compared to continued chemotherapy. In several studies that compared the outcomes of CS and non‐resection, most patients in the non‐resection group did not convert to CS after treatment. For example, in two retrospective studies, only 9/121 (7.4%) and 2/22 (9.1%) patients refused CS.^9^, ^19^ Conversely, many retrospective studies on the efficacy of surgery after preoperative treatment included patients with both borderline resectable and locally advanced PC.^19^, ^20^, ^21^, ^22^, ^23^, ^24^ However, further discussion is needed on whether CS should encompass borderline resectable PC with arterial invasion.^6^ Some retrospective studies evaluated the efficacy of CS in patients with unresectable locally advanced PC after FOLFIRINOX or GnP chemotherapy. The range of median OS from diagnosis or chemotherapy initiation has been reported to be 27.5–56 months.^10^, ^12^, ^20^, ^24^, ^25^ In line with these results, our study highlights the promising efficacy of CS.

Although this study demonstrated that the OS of the surgery group was longer than that of the control group with an HR of 0.47, the Kaplan–Meier curve crossed the mark at 12 months, indicating that the proportional hazard assumption may not have been met, and the interpretation of the hazard ratios obtained in Cox regression might need to include the time‐dependent effects and potential differences between the subgroups. The prognosis of the exploratory laparotomy only group was extremely poor, with a median OS of 7.1 months. One reason for the poor prognosis could be that these patients were likely to discontinue chemotherapy pre‐ and post‐surgery, despite an unresectable status. This study also investigated the differences between the resection and exploratory laparotomy only groups. A difference was observed between the groups in the time until laparotomy from the imaging date. The proportion of patients with ≥4 weeks until laparotomy from decision of potentially resectable PC was higher than that in the only exploratory laparotomy group than in the resection group. Therefore, the duration of CS from completion of chemotherapy may be as short as possible if physical condition and laboratory tests such as bone marrow function permit surgery, because PC is an extremely aggressive disease. Currently, the decision for CS is based on imaging and tumor markers such as CA 19‐9 and the consensus of a multidisciplinary team. However, we found that these modalities were insufficient to predict the success of CS before laparotomy in the resection and exploratory laparotomy only groups, indicating the need for biomarkers to identify patients who can benefit from CS. Circulating tumor DNA liquid biopsy may aid in the diagnosis of occult metastasis and monitoring the response to preoperative chemotherapy.^26^

CS for locally advanced PC is occasionally performed with combined resection of the artery. In our study, approximately 20% of the patients underwent distal pancreatectomy with celiac axis resection. This study demonstrated that the safety of CS was within the acceptable range, with Clavien‐Dindo ≥IIIa postoperative complication and in‐hospital mortality rates of 19.6% and 0.5%, respectively, despite it being an aggressive procedure.

Notably, the multivariate analysis revealed that chemotherapy duration was not associated with OS. A retrospective study of unresectable PC resected after chemotherapy demonstrated a favorable prognosis achieved by CS at ≥8 months after chemotherapy induction;^27^ however, this study was conducted before the FOLFIRINOX or GnP era. Current PC treatments have undergone marked advancements since the introduction of FOLFIRINOX and GnP chemotherapy. However, both FOLFIRINOX and GnP chemotherapy may be more toxic than gemcitabine monotherapy. Most patients treated with these regimens cannot continue oxaliplatin or nab‐paclitaxel for 8 months because of peripheral neuropathy. As expected, the median chemotherapy duration was approximately 6 months in the entire cohort of our study. Although there is no consensus on the optimal duration from preoperative chemotherapy induction to CS,^6^ no association has been demonstrated between chemotherapy duration and OS in other studies, which is consistent with our findings.^21^, ^23^, ^24^ Therefore, the optimal preoperative chemotherapy duration should be individualized according to factors such as imaging and biomarker data and patients' physical condition.

Our study also revealed that the extent of disease at diagnosis, whether locally advanced or metastatic, did not affect the OS. This finding was similar to that of a previous retrospective study on primary tumor resection in patients with “disappearance” of liver metastasis after chemotherapy, which reported a median OS of 56 months from diagnosis.^8^ However, our results should not be overinterpreted because our cohort did not include patients with clinical and pathological M1 disease, such as those who had undergone resection of oligometastasis or positive peritoneal lavage cytology at CS. A retrospective study on CS for pathologically M0 status after preoperative chemotherapy in patients with initially metastatic PC demonstrated encouraging median OS of 25.5 months after resection, but the median OS of CS for pathologically M1 status was only 10.7 months.^28^ Another study reported that CS for M1 disease demonstrated promising efficacy with a median OS of 21.9 months.^29^ Therefore, further research with a large sample size is needed to evaluate the efficacy of CS in patients with metastatic lesions.

Multivariate analysis revealed that FOLFIRINOX was associated with favorable prognosis. A post hoc analysis revealed possible reasons for this finding. First, the pathological response of Evans grade III or IV was higher in the FOLFIRINOX group (31.4%) than that in the GnP group (18.9%). Several studies have reported an association between pathological response and survival in patients with resected PC.^20^, ^23^ Additionally, some retrospective studies have revealed that the pathological response to FOLFIRINOX is higher than that to GnP.^30^, ^31^ However, these findings need to be interpreted with caution. To date, only one randomized phase II trial, JCOG1407, has compared modified FOLFIRINOX and GnP in chemotherapy‐naïve patients with locally advanced PC. The 1‐year OS was similar at 77.4% and 82.5% for modified FOLFIRINOX and GnP, respectively. Moreover, approximately 8% of the patients in both arms who responded positively to chemotherapy underwent CS.^32^ In our study, the entire cohort achieved a potentially resectable status with an excellent response to FOLFIRINOX or GnP chemotherapy. Although patients with homologous recombination deficiency respond to platinum‐containing regimens,^33^ this information was not collected in our study. Second, the proportion of arterial invasion based on pre‐CS imaging was higher in the GnP group than that in the FOLFIRINOX group. Therefore, this finding cannot be used to recommend the selection of a first‐line chemotherapy regimen, whether FOLFIRINOX or GnP.

Multivariate analysis further revealed that the OS of the female patients was longer than that of the male patients. Generally, women live longer than men do, and the definition of OS in our study was not disease specific. Although we did not collect the causes of death, as the survival duration increased, the number of deaths due to reasons other than PC also increased.

This study had some limitations. First, it was retrospective with a small control group. The validity of the enrollment could not be confirmed because this study could not show data on the total number of patients receiving chemotherapy as locally advanced or metastatic PC at the participating centers during the study period, along with the percentage of these patients deemed eligible for CS. However, the study results are reflective of real‐world clinical practice. Patients with an initially unresectable PC status that converted to a potentially resectable status after chemotherapy were unlikely to refuse CS. Notably, the control group included only 10 patients. Therefore, the possibility of a randomized controlled study comparing the surgery and control groups was very low, and our study design was acceptable. Second, the resection margin was decided according to the institutional policy. Third, the interval from imaging of response to chemotherapy and postoperative follow‐up was based on the treating physicians' discretion, which may have affected the chemotherapy duration and RFS; they did not affect the primary endpoint of our study, that is, OS from the date of imaging. Fourth, tumors in the control group were judged as resectable solely based on imaging assessments, whereas in the surgery group, tumors diagnosed as resectable via imaging were further subjected to surgical and pathological confirmation, with exclusion criteria including patients with confirmed distant metastases such as liver metastases, para‐aortic lymph node metastases, and positive peritoneal lavage cytology. The comparison analysis between these two groups introduces bias into this study. Finally, the study excluded cases involving resection of metastatic lesions during CS, while including the 13 patients who underwent exploratory laparotomy only.

In conclusion, CS can have promising outcomes in patients with initially unresectable PC deemed potentially resectable after sufficient FOLFIRINOX or GnP chemotherapy regardless of its duration. Future prospective multinational collaborative studies should confirm the efficacy of CS, the optimal duration of preoperative chemotherapy, and the optimal regimens.

## FUNDING INFORMATION

This study was supported by the Federation of Asian Clinical Oncology.

## CONFLICT OF INTEREST STATEMENT

NO has received personal fees from AstraZeneca. UM has received grants from Taiho Pharmaceutical, AstraZeneca, MSD, Nihon Servier, Ono Pharmaceutical, Incyte Biosciences Japan, Chugai Pharmaceutical, Boehringer Ingelheim, J‐Pharma, Eisai, Novartis Pharma, Astellas Pharma, Delta‐Fly‐Pharma, Novocure, and Chiome Bioscience, and personal fees from Taiho Pharmaceutical. MO has received grants from Taiho Pharmaceutical. KY has received grants from Chugai Pharmaceutical. HY has received grants from Taiho Pharmaceutical, and the endowed course by Yakult Honsha. JF has received grants from MSD, J‐Pharma, Delta‐Fly‐Pharma, Taiho Pharmaceutical, Eisai, and AstraZeneca, and personal fees from Ono Pharmaceutical, Chugai Pharmaceutical, AstraZeneca, and Incyte Biosciences Japan. The other authors have no conflicts of interest to declare.

## Supporting information


Appendix S1.

## Data Availability

The data underlying this article will be shared on reasonable request to the corresponding author.
