# Supplementary material for: Outcomes of patients with initially unresectable pancreatic cancer who underwent conversion surgery after FOLFIRINOX or gemcitabine plus nab‐paclitaxel chemotherapy: A multicenter retrospective cohort study (PC‐CURE‐1)
Source: J Hepatobiliary Pancreat Sci. 2024 Aug 16;31(11):816–29. doi: 10.1002/jhbp.12066 (PMC11589395; doi:10.1002/jhbp.12066)

**Supplemental Tables**

Table S1. Summary of the exploratory laparotomy only group

| Patient | Extent of disease | Reasons for exploratory laparotomy only | | | | | |
| --- | --- | --- | --- | --- | --- | --- | --- |
|  |  | Locally advanced | Liver metastasis | Paraaortic lymph node metastasis | Peritoneal metastasis | Others | Specifics |
| 1 | M |  | Yes |  |  |  |  |
| 2 | M |  |  | Yes |  |  |  |
| 3 | LA |  |  |  | Yes |  |  |
| 4 | LA |  |  | Yes |  |  |  |
| 5 | M |  |  |  | Yes |  |  |
| 6 | M |  |  | Yes |  |  |  |
| 7 | LA | Yes |  |  |  |  |  |
| 8 | LA |  |  |  | Yes |  |  |
| 9 | LA |  | Yes |  | Yes |  |  |
| 10 | LA |  | Yes |  |  |  |  |
| 11 | LA |  |  |  |  | Yes | Proper hepatic artery invasion |
| 12 | LA | Yes |  |  |  |  |  |
| 13 | LA |  |  |  |  | Yes | Cytology positive |

Abbreviations: M, metastatic disease, LA, locally advanced disease

Table S2. Multivariate analysis of the overall survival of the surgery group divided into 4-month periods of chemotherapy

|  | Hazard ratio (95% CI) | *P* value |
| --- | --- | --- |
| Sex |  |  |
| Male vs. Female | 1.57 (1.06, 2.31) | 0.022 |
| Age |  |  |
| <65 years vs. ≥65 years | 1.30 (0.89, 1.95) | 0.201 |
| ECOG PS |  |  |
| 0 vs. 1 or 2 | 1.06 (0.62, 1.84) | 0.821 |
| Extent of disease |  |  |
| Locally advanced vs. metastatic | 0.82 (0.45, 1.51) | 0.529 |
| Tumor location |  |  |
| Head vs. Body or tail | 0.84 (0.57, 1.25) | 0.395 |
| Tumor diameter prior to chemotherapy | 1.00 (0.98, 1.01) | 0.947 |
| Regional lymph node metastasis prior to chemotherapy |  |  |
| Yes vs. no | 0.90 (0.58, 1.91) | 0.649 |
| Arterial invasion prior to chemotherapy |  |  |
| Yes vs. no | 1.06 (0.58, 1.91) | 0.859 |
| Portal vein invasion prior to chemotherapy |  |  |
| Yes vs. no | 1.21 (0.79, 1.85) | 0.387 |
| Chemotherapy |  |  |
| FOLFIRINOX vs. GnP | 0.38 (0.22, 0.65) | <0.001 |
| RECIST version 1.1 |  |  |
| CR/PR vs. SD | 0.56 (0.36, 0.88) | 0.012 |
| Chemotherapy duration |  |  |
| <4 months vs. ≥4 months | 0.87 (0.35, 2.15) | 0.763 |
| Radiotherapy |  |  |
| None vs. performed | 1.23 (0.71, 2.11) | 0.459 |
| CEA prior to chemotherapy |  |  |
| Normal vs. abnormal | 1.14 (0.75, 1.75) | 0.538 |
| CA 19-9 prior to chemotherapy |  |  |
| Normal vs. abnormal | 0.82 (0.46, 1.45) | 0.488 |

Abbreviations: CI, confidence interval; ECOG PS, Eastern Cooperative Oncology Group performance status; FOLFIRINOX, 5-fluorouracil, leucovorin, irinotecan, and oxaliplatin; GnP, gemcitabine plus nab-paclitaxel; RECIST, Response Evaluation Criteria in Solid Tumors; CR, complete response; PR, partial response; SD, stable disease; CEA, serum carcinoembryonic antigen; CA 19-9, serum carbohydrate antigen 19-9

Table S3. Multivariate analysis of the overall survival of the surgery group divided into 8-month periods of chemotherapy

|  | Hazard ratio (95% CI) | *P* value |
| --- | --- | --- |
| Sex |  |  |
| Male vs. Female | 1.60 (1.08, 2.35) | 0.018 |
| Age |  |  |
| <65 years vs. ≥65 years | 1.29 (0.86, 1.93) | 0.225 |
| ECOG PS |  |  |
| 0 vs. 1 or 2 | 1.05 (0.61, 1.82) | 0.850 |
| Extent of disease |  |  |
| Locally advanced vs. metastatic | 0.82 (0.45, 1.50) | 0.520 |
| Tumor location |  |  |
| Head vs. Body or tail | 0.84 (0.57, 1.25) | 0.398 |
| Tumor diameter prior to chemotherapy | 1.00 (0.98, 1.01) | 0.978 |
| Regional lymph node metastasis prior to chemotherapy |  |  |
| Yes vs. no | 0.90 (0.58, 1.40) | 0.647 |
| Arterial invasion prior to chemotherapy |  |  |
| Yes vs. no | 1.05 (0.58, 1.90) | 0.884 |
| Portal vein invasion prior to chemotherapy |  |  |
| Yes vs. no | 1.23 (0.80, 1.89) | 0.345 |
| Chemotherapy |  |  |
| FOLFIRINOX vs. GnP | 0.38 (0.22, 0.65) | <0.001 |
| RECIST version 1.1 |  |  |
| CR/PR vs. SD | 0.57 (0.36, 0.89) | 0.014 |
| Chemotherapy duration |  |  |
| <8 months vs. ≥8 months | 0.68 (0.29, 1.59) | 0.372 |
| Radiotherapy |  |  |
| None vs. performed | 1.24 (0.72, 2.13) | 0.434 |
| CEA prior to chemotherapy |  |  |
| Normal vs. abnormal | 1.12 (0.73, 1.71) | 0.618 |
| CA 19-9 prior to chemotherapy |  |  |
| Normal vs. abnormal | 0.94 (0.51, 1.75) | 0.848 |

Abbreviations: CI, confidence interval; ECOG PS, Eastern Cooperative Oncology Group performance status; FOLFIRINOX, 5-fluorouracil, leucovorin, irinotecan, and oxaliplatin; GnP, gemcitabine plus nab-paclitaxel; RECIST, Response Evaluation Criteria in Solid Tumors; CR, complete response; PR, partial response; SD, stable disease; CEA, serum carcinoembryonic antigen; CA 19-9, serum carbohydrate antigen 19-9

Table S4. Multivariate analysis of the overall survival of the surgery group divided into 4-month periods of chemotherapy in locally advanced pancreatic cancer

|  | Hazard ratio (95% CI) | *P* value |
| --- | --- | --- |
| Sex |  |  |
| Male vs. Female | 1.67 (1.03, 2.72) | 0.038 |
| Age |  |  |
| <65 years vs. ≥65 years | 1.02 (0.63, 1.66) | 0.934 |
| ECOG PS |  |  |
| 0 vs. 1 or 2 | 0.79 (0.41, 1.53) | 0.488 |
| Tumor location |  |  |
| Head vs. Body or tail | 0.72 (0.44, 1.18) | 0.192 |
| Tumor diameter prior to chemotherapy | 0.99 (0.97, 1.01) | 0.506 |
| Regional lymph node metastasis prior to chemotherapy |  |  |
| Yes vs. no | 0.90 (0.58, 1.91) | 0.649 |
| Arterial invasion prior to chemotherapy |  |  |
| Yes vs. no | 0.72 (0.41, 1.24) | 0.236 |
| Portal vein invasion prior to chemotherapy |  |  |
| Yes vs. no | 1.00 (0.63, 1.61) | 0.986 |
| Chemotherapy |  |  |
| FOLFIRINOX vs. GnP | 0.39 (0.19, 0.81) | 0.011 |
| RECIST version 1.1 |  |  |
| CR/PR vs. SD | 0.76 (0.45, 1.30) | 0.317 |
| Chemotherapy duration |  |  |
| <4 months vs. ≥4 months | 1.46 (0.39, 5.50) | 0.574 |
| Radiotherapy |  |  |
| None vs. performed | 1.16 (0.66, 2.04) | 0.608 |
| CEA prior to chemotherapy |  |  |
| Normal vs. abnormal | 0.91 (0.54, 1.54) | 0.723 |
| CA 19-9 prior to chemotherapy |  |  |
| Normal vs. abnormal | 0.58 (0.28, 1.21) | 0.147 |

Abbreviations: CI, confidence interval; ECOG PS, Eastern Cooperative Oncology Group performance status; FOLFIRINOX, 5-fluorouracil, leucovorin, irinotecan, and oxaliplatin; GnP, gemcitabine plus nab-paclitaxel; RECIST, Response Evaluation Criteria in Solid Tumors; CR, complete response; PR, partial response; SD, stable disease; CEA, serum carcinoembryonic antigen; CA 19-9, serum carbohydrate antigen 19-9

Table S5. Multivariate analysis of the overall survival of the surgery group divided into 4-month periods of chemotherapy in metastatic pancreatic cancer

|  | Hazard ratio (95% CI) | *P* value |
| --- | --- | --- |
| Sex |  |  |
| Male vs. Female | 1.51 (0.67, 3.42) | 0.321 |
| Age |  |  |
| <65 years vs. ≥65 years | 1.91 (0.79, 4.59) | 0.149 |
| ECOG PS |  |  |
| 0 vs. 1 or 2 | 3.76 (0.66, 21.4) | 0.135 |
| Tumor location |  |  |
| Head vs. Body or tail | 0.84 (0.33, 2.14) | 0.721 |
| Tumor diameter prior to chemotherapy | 0.99 (0.96, 1.03) | 0.748 |
| Regional lymph node metastasis prior to chemotherapy |  |  |
| Yes vs. no | 0.98 (0.40, 2.41) | 0.969 |
| Arterial invasion prior to chemotherapy |  |  |
| Yes vs. no | 1.49 (0.70, 3.15) | 0.299 |
| Portal vein invasion prior to chemotherapy |  |  |
| Yes vs. no | 2.78 (0.90, 8.57) | 0.075 |
| Chemotherapy |  |  |
| FOLFIRINOX vs. GnP | 0.17 (0.06, 0.49) | 0.001 |
| RECIST version 1.1 |  |  |
| CR/PR vs. SD | 0.27 (0.10, 0.77) | 0.015 |
| Chemotherapy duration |  |  |
| <4 months vs. ≥4 months | 0.62 (0.15, 2.50) | 0.502 |
| Radiotherapy |  |  |
| None vs. performed | 2.21 (0.14, 33.8) | 0.569 |
| CEA prior to chemotherapy |  |  |
| Normal vs. abnormal | 1.66 (0.77, 3.60) | 0.198 |
| CA 19-9 prior to chemotherapy |  |  |
| Normal vs. abnormal | 1.16 (0.41, 3.32) | 0.782 |

Abbreviations: CI, confidence interval; ECOG PS, Eastern Cooperative Oncology Group performance status; FOLFIRINOX, 5-fluorouracil, leucovorin, irinotecan, and oxaliplatin; GnP, gemcitabine plus nab-paclitaxel; RECIST, Response Evaluation Criteria in Solid Tumors; CR, complete response; PR, partial response; SD, stable disease; CEA, serum carcinoembryonic antigen; CA 19-9, serum carbohydrate antigen 19-9

Table S6. Multivariate analysis of the overall survival of the surgery group divided into 6-month periods of chemotherapy in locally advanced pancreatic cancer

|  | Hazard ratio (95% CI) | *P* value |
| --- | --- | --- |
| Sex |  |  |
| Male vs. Female | 1.71 (1.05, 2.80) | 0.031 |
| Age |  |  |
| <65 years vs. ≥65 years | 1.01 (0.62, 1.64) | 0.979 |
| ECOG PS |  |  |
| 0 vs. 1 or 2 | 0.78 (0.40, 1.50) | 0.449 |
| Tumor location |  |  |
| Head vs. Body or tail | 0.75 (0.46, 1.23) | 0.253 |
| Tumor diameter prior to chemotherapy | 0.99 (0.97, 1.01) | 0.523 |
| Regional lymph node metastasis prior to chemotherapy |  |  |
| Yes vs. no | 0.71 (0.40, 1.24) | 0.225 |
| Arterial invasion prior to chemotherapy |  |  |
| Yes vs. no | 0.64 (0.24, 1.71) | 0.376 |
| Portal vein invasion prior to chemotherapy |  |  |
| Yes vs. no | 1.00 (0.62, 1.61) | 0.999 |
| Chemotherapy |  |  |
| FOLFIRINOX vs. GnP | 0.39 (0.19, 0.81) | 0.012 |
| RECIST version 1.1 |  |  |
| CR/PR vs. SD | 0.79 (0.46, 1.35) | 0.387 |
| Chemotherapy duration |  |  |
| <6 months vs. ≥6 months | 0.56 (0.16, 1.99) | 0.370 |
| Radiotherapy |  |  |
| None vs. performed | 1.23 (0.69, 2.19) | 0.475 |
| CEA prior to chemotherapy |  |  |
| Normal vs. abnormal | 0.86 (0.50, 1.47) | 0.570 |
| CA 19-9 prior to chemotherapy |  |  |
| Normal vs. abnormal | 0.77 (0.36, 1.62) | 0.782 |

Abbreviations: CI, confidence interval; ECOG PS, Eastern Cooperative Oncology Group performance status; FOLFIRINOX, 5-fluorouracil, leucovorin, irinotecan, and oxaliplatin; GnP, gemcitabine plus nab-paclitaxel; RECIST, Response Evaluation Criteria in Solid Tumors; CR, complete response; PR, partial response; SD, stable disease; CEA, serum carcinoembryonic antigen; CA 19-9, serum carbohydrate antigen 19-9

Table S7. Multivariate analysis of the overall survival of the surgery group divided into 6-month periods of chemotherapy in metastatic pancreatic cancer

|  | Hazard ratio (95% CI) | *P* value |
| --- | --- | --- |
| Sex |  |  |
| Male vs. Female | 1.52 (0.67, 3.46) | 0.315 |
| Age |  |  |
| <65 years vs. ≥65 years | 1.88 (0.76, 4.67) | 0.171 |
| ECOG PS |  |  |
| 0 vs. 1 or 2 | 3.78 (0.66, 21.7) | 0.135 |
| Tumor location |  |  |
| Head vs. Body or tail | 0.84 (0.33, 2.15) | 0.718 |
| Tumor diameter prior to chemotherapy | 1.00 (0.96, 1.03) | 0.773 |
| Regional lymph node metastasis prior to chemotherapy |  |  |
| Yes vs. no | 1.01 (0.41, 2.51) | 0.976 |
| Arterial invasion prior to chemotherapy |  |  |
| Yes vs. no | 1.51 (0.71, 3.21) | 0.282 |
| Portal vein invasion prior to chemotherapy |  |  |
| Yes vs. no | 2.76 (0.90, 8.51) | 0.076 |
| Chemotherapy |  |  |
| FOLFIRINOX vs. GnP | 0.17 (0.06, 0.50) | 0.001 |
| RECIST version 1.1 |  |  |
| CR/PR vs. SD | 0.29 (0.10, 0.80) | 0.017 |
| Chemotherapy duration |  |  |
| <6 months vs. ≥6 months | 0.79 (0.19, 3.33) | 0.746 |
| Radiotherapy |  |  |
| None vs. performed | 2.17 (0.14, 33.5) | 0.579 |
| CEA prior to chemotherapy |  |  |
| Normal vs. abnormal | 1.73 (0.80, 3.72) | 0.163 |
| CA 19-9 prior to chemotherapy |  |  |
| Normal vs. abnormal | 1.04 (0.32, 3.38) | 0.943 |

Abbreviations: CI, confidence interval; ECOG PS, Eastern Cooperative Oncology Group performance status; FOLFIRINOX, 5-fluorouracil, leucovorin, irinotecan, and oxaliplatin; GnP, gemcitabine plus nab-paclitaxel; RECIST, Response Evaluation Criteria in Solid Tumors; CR, complete response; PR, partial response; SD, stable disease; CEA, serum carcinoembryonic antigen; CA 19-9, serum carbohydrate antigen 19-9

Table S8. Multivariate analysis of the overall survival of the surgery group divided into 8-month periods of chemotherapy in locally advanced pancreatic cancer

|  | Hazard ratio (95% CI) | *P* value |
| --- | --- | --- |
| Sex |  |  |
| Male vs. Female | 1.76 (1.08, 2.88) | 0.315 |
| Age |  |  |
| <65 years vs. ≥65 years | 0.96 (0.59, 1.57) | 0.870 |
| ECOG PS |  |  |
| 0 vs. 1 or 2 | 0.77 (0.40, 1.48) | 0.439 |
| Tumor location |  |  |
| Head vs. Body or tail | 0.73 (0.45, 1.18) | 0.200 |
| Tumor diameter prior to chemotherapy | 0.99 (0.97, 1.01) | 0.523 |
| Regional lymph node metastasis prior to chemotherapy |  |  |
| Yes vs. no | 0.69 (0.39, 1.21) | 0.195 |
| Arterial invasion prior to chemotherapy |  |  |
| Yes vs. no | 0.61 (0.23, 1.63) | 0.326 |
| Portal vein invasion prior to chemotherapy |  |  |
| Yes vs. no | 1.03 (0.64, 1.67) | 0.889 |
| Chemotherapy |  |  |
| FOLFIRINOX vs. GnP | 0.39 (0.18, 0.81) | 0.011 |
| RECIST version 1.1 |  |  |
| CR/PR vs. SD | 0.82 (0.48, 1.40) | 0.463 |
| Chemotherapy duration |  |  |
| <8 months vs. ≥8 months | 0.34 (0.10, 1.20) | 0.094 |
| Radiotherapy |  |  |
| None vs. performed | 1.22 (0.70, 2.15) | 0.482 |
| CEA prior to chemotherapy |  |  |
| Normal vs. abnormal | 0.83 (0.49, 1.42) | 0.498 |
| CA 19-9 prior to chemotherapy |  |  |
| Normal vs. abnormal | 0.94 (0.45, 1.97) | 0.867 |

Abbreviations: CI, confidence interval; ECOG PS, Eastern Cooperative Oncology Group performance status; FOLFIRINOX, 5-fluorouracil, leucovorin, irinotecan, and oxaliplatin; GnP, gemcitabine plus nab-paclitaxel; RECIST, Response Evaluation Criteria in Solid Tumors; CR, complete response; PR, partial response; SD, stable disease; CEA, serum carcinoembryonic antigen; CA 19-9, serum carbohydrate antigen 19-9

Table S9. Multivariate analysis of the overall survival of the surgery group divided into 8-month periods of chemotherapy in metastatic pancreatic cancer

|  | Hazard ratio (95% CI) | *P* value |
| --- | --- | --- |
| Sex |  |  |
| Male vs. Female | 1.50 (0.66, 3.43) | 0.333 |
| Age |  |  |
| <65 years vs. ≥65 years | 1.77 (0.72, 4.35) | 0.212 |
| ECOG PS |  |  |
| 0 vs. 1 or 2 | 3.90 (0.68, 22.5) | 0.128 |
| Tumor location |  |  |
| Head vs. Body or tail | 0.83 (0.32, 2.14) | 0.707 |
| Tumor diameter prior to chemotherapy | 1.00 (0.96, 1.03) | 0818 |
| Regional lymph node metastasis prior to chemotherapy |  |  |
| Yes vs. no | 1.00 (0.40, 2.48) | 0.995 |
| Arterial invasion prior to chemotherapy |  |  |
| Yes vs. no | 1.50 (0.71, 3.20) | 0.290 |
| Portal vein invasion prior to chemotherapy |  |  |
| Yes vs. no | 2.80 (0.91, 8.59) | 0.072 |
| Chemotherapy |  |  |
| FOLFIRINOX vs. GnP | 0.18 (0.07, 0.52) | 0.001 |
| RECIST version 1.1 |  |  |
| CR/PR vs. SD | 0.30 (0.11, 0.82) | 0.019 |
| Chemotherapy duration |  |  |
| <8 months vs. ≥8 months | 1.15 (0.27, 4.98) | 0.847 |
| Radiotherapy |  |  |
| None vs. performed | 2.07 (0.13, 32.6) | 0.604 |
| CEA prior to chemotherapy |  |  |
| Normal vs. abnormal | 1.80 (0.84, 3.85) | 0.132 |
| CA 19-9 prior to chemotherapy |  |  |
| Normal vs. abnormal | 0.82 (0.22, 2.98) | 0.761 |

Abbreviations: CI, confidence interval; ECOG PS, Eastern Cooperative Oncology Group performance status; FOLFIRINOX, 5-fluorouracil, leucovorin, irinotecan, and oxaliplatin; GnP, gemcitabine plus nab-paclitaxel; RECIST, Response Evaluation Criteria in Solid Tumors; CR, complete response; PR, partial response; SD, stable disease; CEA, serum carcinoembryonic antigen; CA 19-9, serum carbohydrate antigen 19-9

Table S10. Patient characteristics prior to FOLFIRINOX and GnP

| Characteristics | FOLFIRINOX (*N*=53) | GnP (*N*=154) |
| --- | --- | --- |
| Sex |  |  |
| Male/female, *N* (%) | 26/27 (49.1/50.9) | 85/69 (55.2/44.8) |
| Age, years |  |  |
| Median, (IQR) | 63.0 (55.0–66.0) | 67.0 (61.0–71.0) |
| <65 years/≥65 years, *N* (%) | 34/19 (64.2/35.8) | 56/98 (36.4/63.6) |
| ECOG PS |  |  |
| 0/1/2, *N* (%) | 51/1/1 (96.2/1.9/1.9) | 128 (83.1/16.2/0.6) |
| Country |  |  |
| Japan/China, *N* (%) | 51/2 (96.2/3.8) | 151/3 (98.1/1.9) |
| Extent of disease |  |  |
| Locally advanced/metastatic, *N* (%) | 24/29 (45.3/54.7) | 112/42 (72.7/27.3) |
| Tumor location |  |  |
| Head/body or tail, *N* (%) | 32/21 (60.4/30.9) | 86/68 (55.8/44.2) |
| Tumor diameter, mm |  |  |
| Median (IQR) | 30.0 (23.0–38.0) | 30.0 (25.0–38.0) |
| Regional lymph node metastasis |  |  |
| Yes/no, *N* (%) | 18/35 (34.0/66.0) | 42/112 (27.3/72.7) |
| Biliary drainage |  |  |
| Yes/no, *N* (%) | 20/33 (37.7/62.3) | 51/103 (33.1/66.9) |
| Unresectability factors, *N* (%) |  |  |
| Arterial invasion | 28 (52.8) | 119 (77.3) |
| Portal vein invasion | 26 (49.1) | 78 (50.6) |
| Liver metastasis | 18 (34.0) | 26 (16.9) |
| Lung metastasis | 1 (1.9) | 2 (1.3) |
| Lymph node metastasis | 8 (15.1) | 8 (5.2) |
| Peritoneal metastasis | 6 (11.3) | 8 (5.2) |
| Ascites | 0 | 1 (0.6) |
| Pleural metastasis | 0 | 1 (0.6) |
| Others | 2 (3.8) | 0 |
| TNM by UICC (version 7), *N* (%) |  |  |
| Stage IIA | 4 (7.5) | 10 (6.5) |
| Stage IIB (T3+N1+M0) | 2 (3.8) | 8 (5.2) |
| Stage III | 18 (34.0) | 94 (61.0) |
| Stage IV | 29 (54.7) | 42 (27.3) |
| CEA, ng/mL |  |  |
| Median (IQR) | 3.7 (2.0–5.6) | 3.2 (2.0–6.1) |
| Normal (≤5.0 ng/mL), *N* (%) | 33 (66.0) | 101 (69.7) |
| Abnormal (>5.0 ng/mL), *N* (%) | 17 (34.0) | 44 (30.3) |
| CA 19-9, U/mL |  |  |
| Median (IQR) | 140.6 (32.3–711.2) | 245.1 (50.0–1018.0) |
| Normal (≤37.0 U/mL), *N* (%) | 14 (26.4) | 31 (20.4) |
| Abnormal (>37.0 U/mL), *N* (%) | 39 (73.6) | 121 (79.6) |

Abbreviations: FOLFIRINOX, 5-fluorouracil, leucovorin, irinotecan, and oxaliplatin; GnP, gemcitabine plus nab-paclitaxel; IQR, interquartile range; ECOG PS, Eastern Cooperative Oncology Group performance status; CEA, serum carcinoembryonic antigen; CA 19-9, serum carbohydrate antigen 19-9

Table S11. Pre-operative patient characteristics post FOLFIRINOX and GnP in resected patients

| Characteristics | FOLFIRINOX (*N*=51) | GnP (*N*=143) |
| --- | --- | --- |
| Chemotherapy duration, months |  |  |
| Median (IQR) | 8.6 (5.0–16.1) | 6.5 (4.5–9.1) |
| <6 months/≥6 months, *N* (%) | 15/36 (29.4/70.6) | 64/79 (44.8/55) |
| <8 months/≥8 months, *N* (%) | 24/27 (47.1/52.9) | 93/50 (65.0/35) |
| Duration to surgery from last chemotherapy cycle |  |  |
| <4 weeks/≥4 weeks, *N* (%) | 20/31 (39.2/60.8) | 65/78 (45.5/54.5) |
| Response of chemotherapy |  |  |
| CR/PR/SD, *N* (%) | 0/39/12 (0/76.5/23.5) | 2/107/34 (1.4/74.8/23.8) |
| Radiotherapy, *N* (%) |  |  |
| Performed |  |  |
| Concurrent | 1 (1.9) | 18 (11.7) |
| Sequential | 2 (3.8) | 16 (10.4) |
| None | 48 (94.1) | 11 (76.9) |
| Regional lymph node metastasis |  |  |
| Yes/no, *N* (%) | 9/42 (17.6/82.4) | 25/118 (17.5/82.5) |
| Biliary drainage |  |  |
| Yes/no, *N* (%) | 19/42 (37.3/82.4) | 49/118 (34.3/82.5) |
| TNM by UICC (version 7), *N* (%) |  |  |
| Stage IA | 4 (7.8) | 5 (3.5) |
| Stage IB | 0 | 1 (0.7) |
| Stage IIA | 23 (45.1) | 57 (39.9) |
| Stage IIB (T3+N1+M0) | 9 (17.6) | 14 (9.8) |
| Stage III | 15 (29.4) | 66 (46.2) |
| Arterial invasion |  |  |
| Yes/no, *N* (%) | 27/24 (52.9/47.1) | 110/33 (76.9/23.1) |
| Portal vein invasion |  |  |
| Yes/no, *N* (%) | 24/27 (47.1/52.9) | 53/90 (37.1/62.9) |
| CEA, ng/mL |  |  |
| Median (IQR) | 2.8 (2.2−4.7) | 3.0 (2.0−4.0) |
| Normal (≤5.0 ng/mL), *N* (%) | 44 (86.3) | 123 (86.0) |
| Abnormal (>5.0 ng/mL), *N* (%) | 7 (13.7) | 20 (14.0) |
| CA 19-9, U/mL |  |  |
| Median (range) | 19.0 (10.0−35.8) | 21.0 (8.2−52.1) |
| Normal (≤37.0 U/mL), *N* (%) | 38 (74.5) | 99 (69.2) |
| Abnormal (>37.0 U/mL), *N* (%) | 13 (25.5) | 44 (30.8) |

Abbreviations: FOLFIRINOX, 5-fluorouracil, leucovorin, irinotecan, and oxaliplatin; GnP, gemcitabine plus nab-paclitaxel; IQR, interquartile range; CR, complete response; PR, partial response; SD, stable disease; CEA, serum carcinoembryonic antigen; CA 19-9, serum carbohydrate antigen 19-9

Table S12. Operative information post FOLFIRINOX and GnP

|  | FOLFIRINOX (*N*=51) | GnP (*N*=143) |
| --- | --- | --- |
| Operation type |  |  |
| PD/DP/TP/DP-CAR, *N* (%) | 32/10/0/9  (62.7/19.6/0/17.6) | 81/27/5/30 (56.6/18.9/3.5/21.0) |
| Combined resections of other structures/organs, *N* (%) |  |  |
| None | 22 (43.1) | 58 (40.6) |
| Common hepatic artery | 1 (2.0) | 12 (8.4) |
| Celiac artery | 5 (9.8) | 27 (18.9) |
| SMA | 0 | 0 |
| PV/SMV | 20 (39.2) | 57 (39.9) |
| Liver | 0 | 0 |
| Colon | 3 (5.9) | 3 (2.1) |
| Adrenal | 4 (7.8) | 16 (11.2) |
| Others | 7 (13.7) | 12 (8.4) |
| Pathological findings |  |  |
| R status, N (%) |  |  |
| R0/R1/R2 | 48/3/0 (94.1/5.9/0) | 130/11/2 (90.9/7.7/1.4) |
| TNM by UICC (version 7), *N* (%) |  |  |
| Stage 0 | 1 (2.0) | 2 (1.4) |
| Stage IA | 8 (15.7) | 12 (8.4) |
| Stage IB | 2 (3.9) | 3 (2.1) |
| Stage IIA | 24 (47.1) | 55 (38.5) |
| Stage IIB (T1, T2+N1+M0) | 2 (3.9) | 8 (5.6) |
| Stage IIB (T3+N1+M0) | 9 (17.6) | 46 (32.2) |
| Stage III | 1 (2.0) | 13 (9.1) |
| No residual cancer | 4 (7.8) | 4 (2.8) |
| Evans grading system, *N* (%) |  |  |
| I/IIa/IIb/III/IV/NA | 2/15/16/11/5/2  (3.9/29.4/31.4/21.6/9.8/3.9) | 30/53/30/20/7/3  (21.0/37.1/21.0/14.0/4.9/2.1) |
| Post-operative mortality and morbidity |  |  |
| In-hospital death, *N* (%) | 0 | 1 (0.7) |
| Clavien–Dindo grade ≥Ⅲa, *N* (%) | 9 (17.6) | 32 (22.4) |
| Adjuvant therapy |  |  |
| S-1 | 29 (56.9) | 88 (61.5) |
| Gemcitabine | 0 | 7 (4.9) |
| Gemcitabine plus capecitabine | 3 (5.9) | 22 (15.4) |
| Capecitabine | 1 (2.0) | 0 |
| Others | 6 (11.8) | 2 (1.4) |
| None | 12 (23.5) | 24 (16.8) |

Abbreviations: PD, pancreaticoduodenectomy; DP, distal pancreatectomy; TP, total pancreatectomy

DP-CAR, distal pancreatectomy with celiac axis resection, SMA, superior mesenteric artery; PV, portal vein; SMV, superior mesenteric vein

**Figure S1.** Kaplan–Meier curves of overall survival from the day when initially locally advanced pancreatic cancer was determined as potentially curative resection on images

**
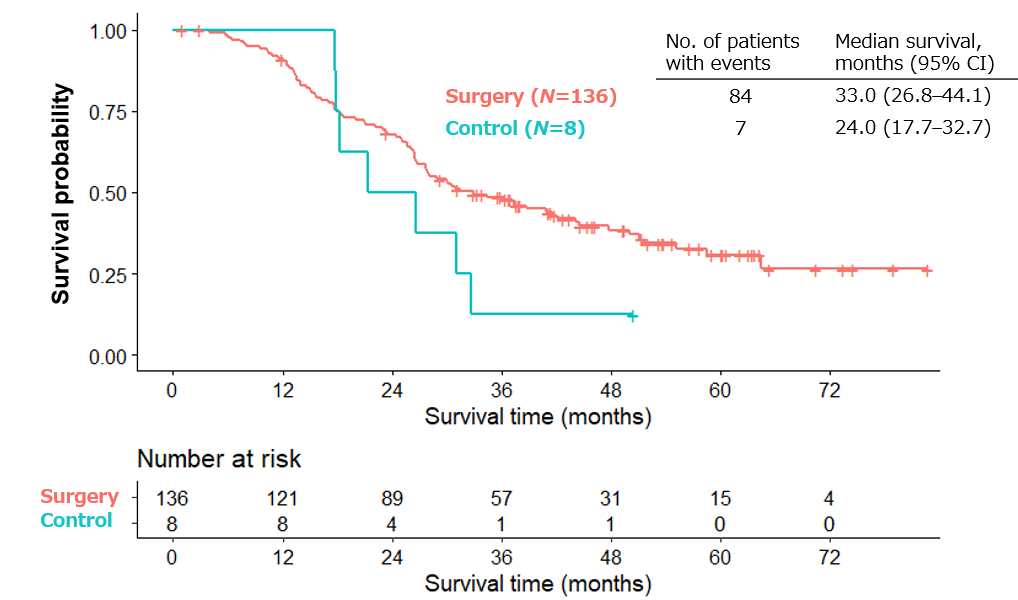
**

**Figure S2.** Kaplan–Meier curves of overall survival from the day when initially metastatic pancreatic cancer was determined as potentially curative resection on images

**
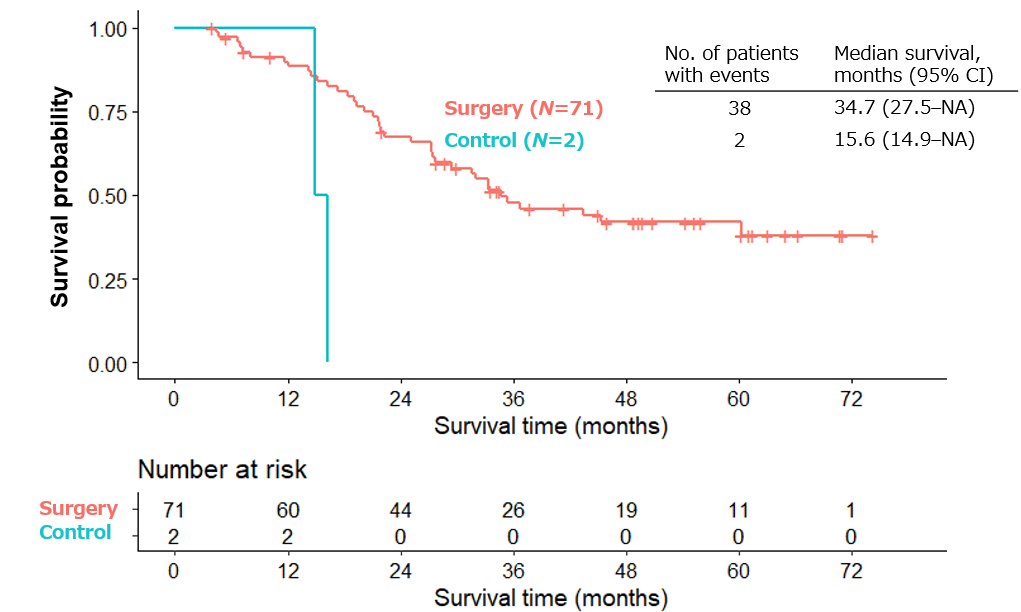
**

**Figure S3.** Kaplan–Meier curve for relapse-free survival in locally advanced pancreatic cancer

**
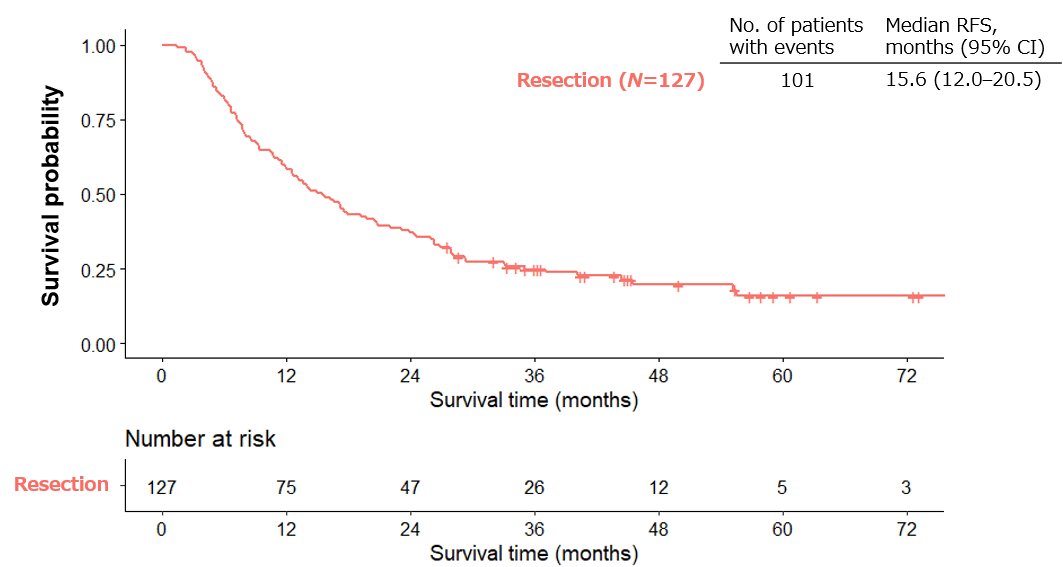
**

**Figure S4.** Kaplan–Meier curve for relapse-free survival in metastatic pancreatic cancer

**
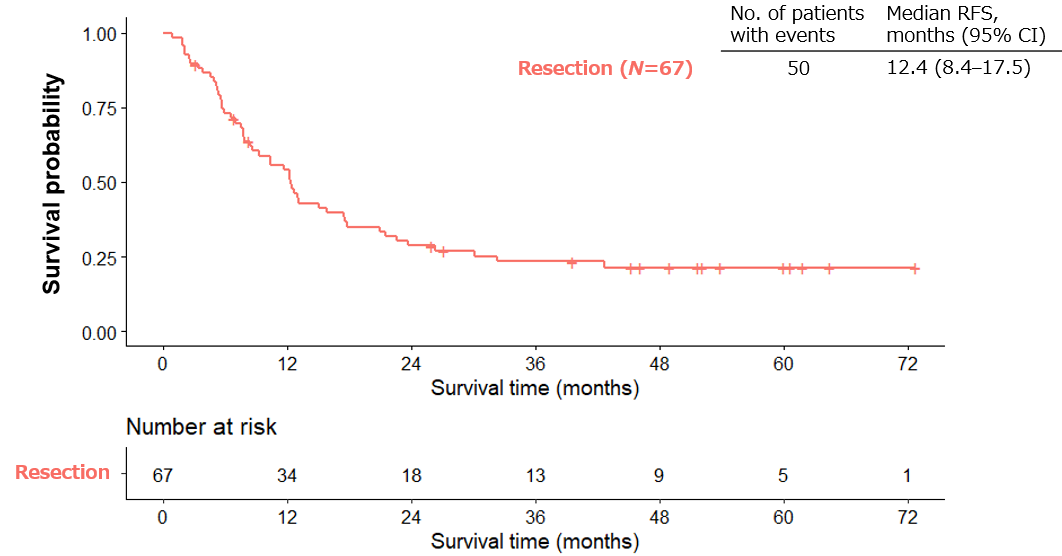
**

**Figure S5.** Kaplan–Meier curves for survival from laparotomy in locally advanced pancreatic cancer


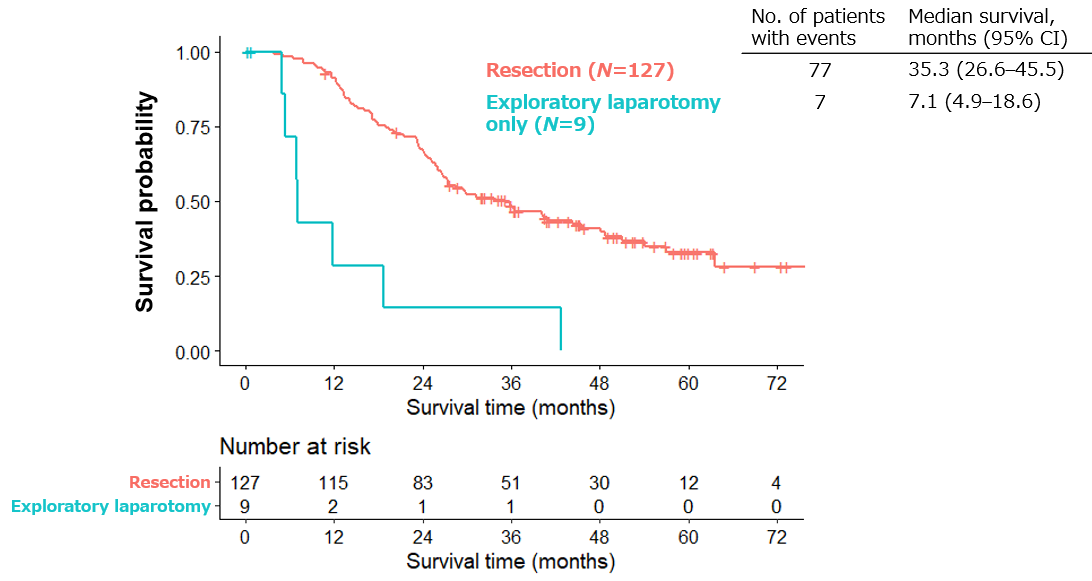


**Figure S6.** Kaplan–Meier curves for survival from laparotomy in metastatic pancreatic cancer

**
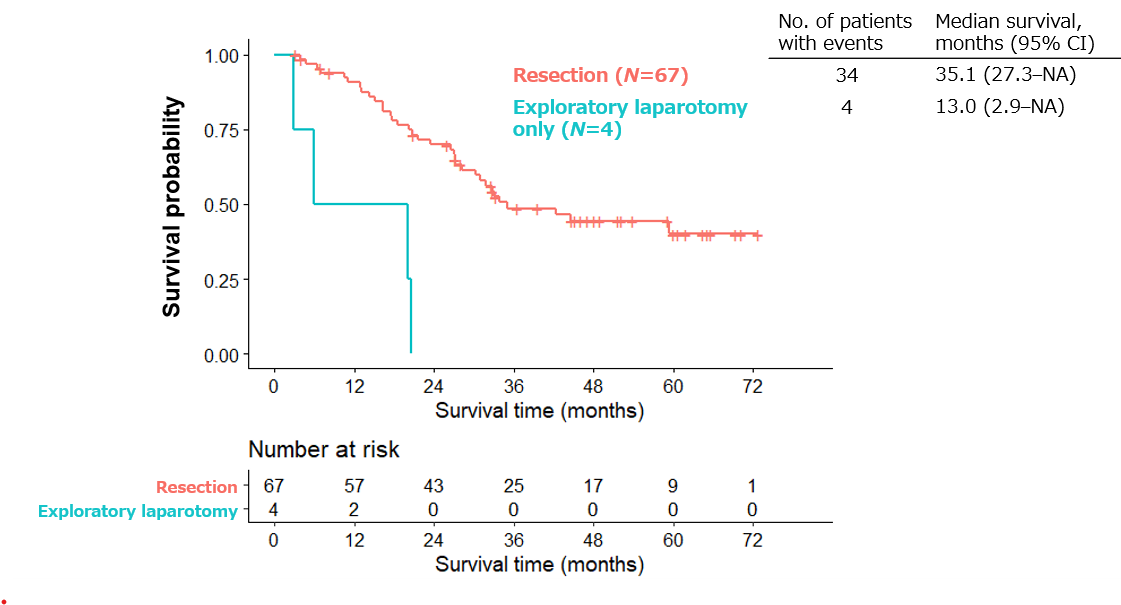
**

**Figure S7.** Kaplan–Meier curves of FOLFIRINOX and GnP for overall survival from the day when initially unresectable pancreatic cancer was diagnosed as potentially curative resection by imaging


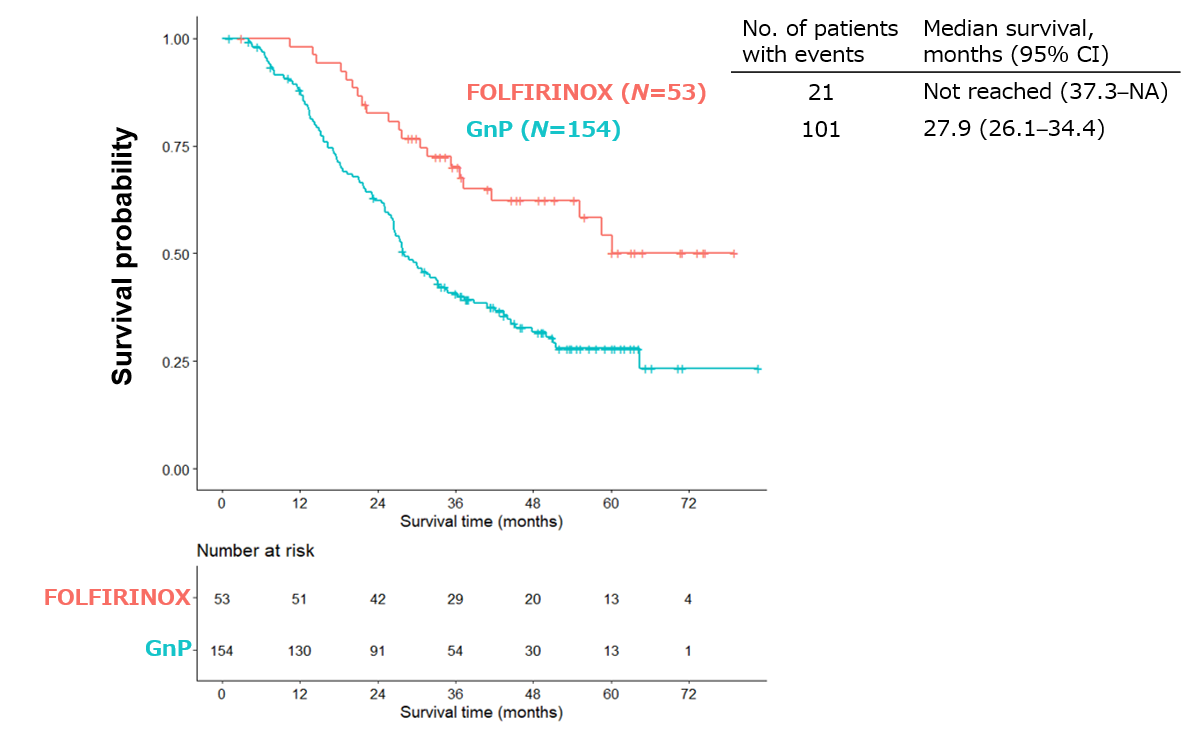


**Figure S8.** Kaplan–Meier curves of FOLFIRINOX and GnP for relapse-free survival


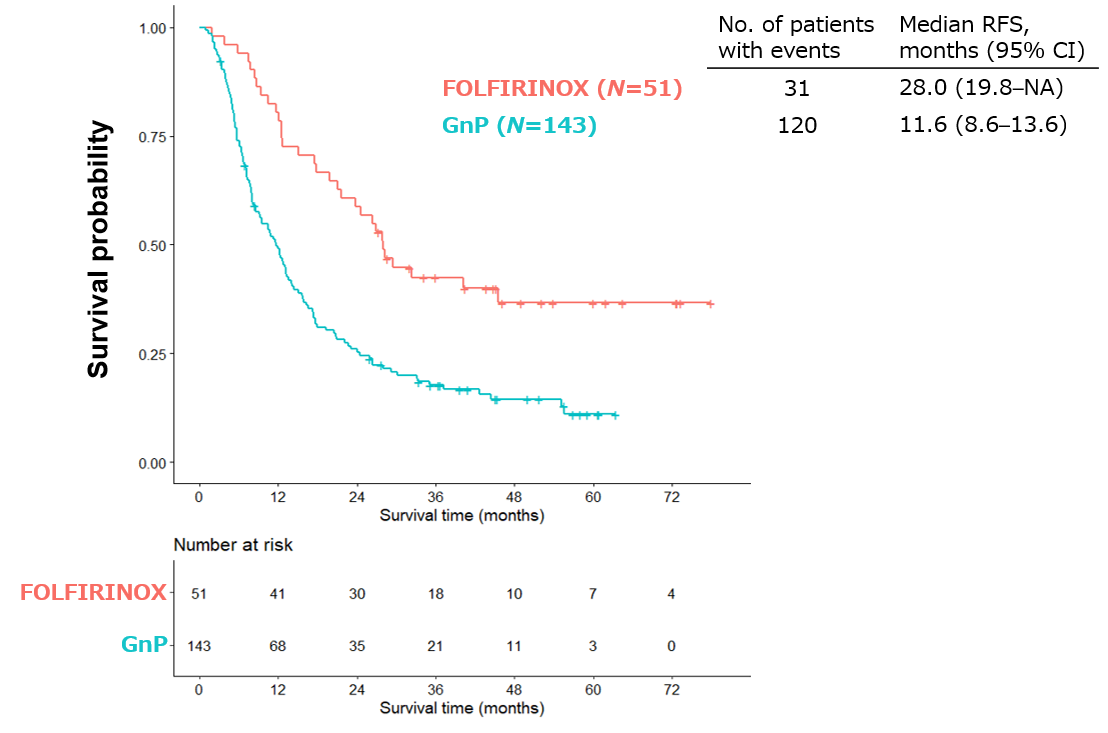


**Figure S9.** Kaplan–Meier curves of FOLFIRINOX and GnP for post-resection survival


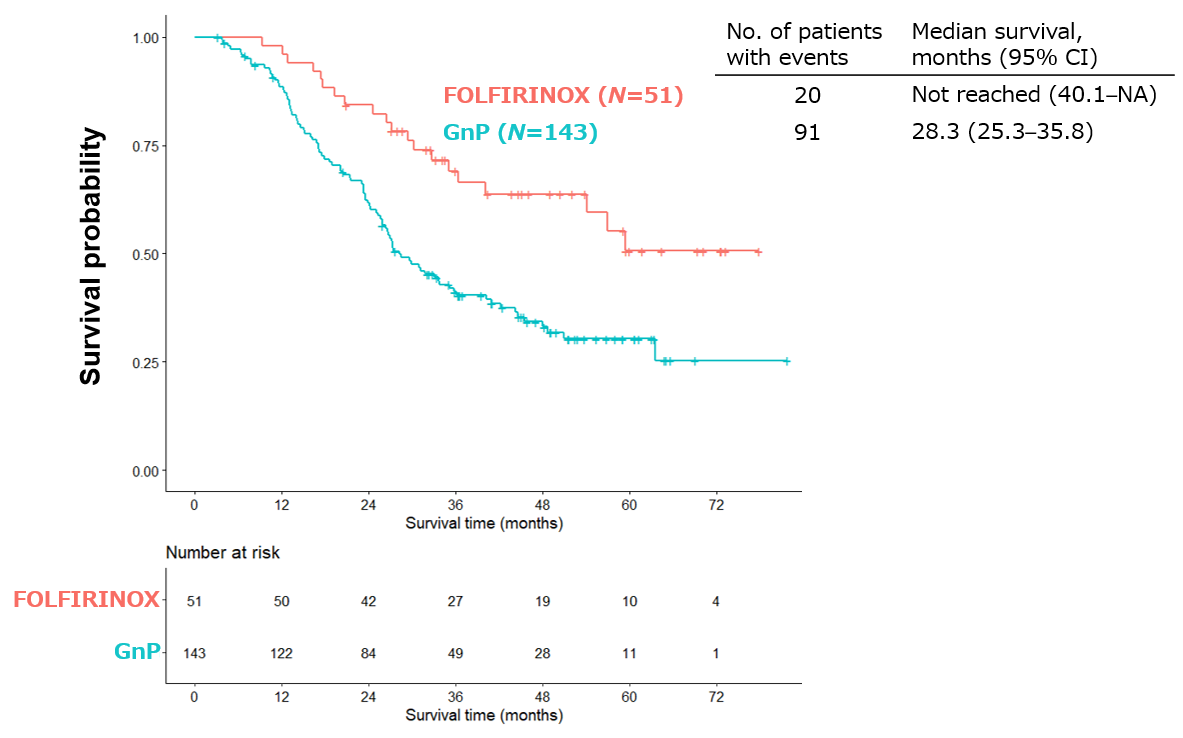

Supplement: Supplementary file 1 — Appendix S1. [file JHBP-31-816-s001.docx]
